# Investigating the impact of drill material on hole quality in jute/palm fiber reinforced hybrid composite drilling with uncertainty analysis

**DOI:** 10.1016/j.heliyon.2024.e36925

**Published:** 2024-08-26

**Authors:** Mohamed Slamani, Abdelmalek Elhadi, Salah Amroune, Mustapha Arslane, Walid Jomaa, Hassan Fouad, Jean-François Chatelain, Mohammad Jawaid

**Affiliations:** a Laboratory of Materials and Mechanics of Structures (LMMS), Faculty of Technology, University of M'sila, PO. Box. 166 Ichebilia, 28000, M'sila, Algeria; b Mechanical Engineering Department, École de Technologie Supérieure, 1100 R. Notre Dame O, Montreal, QC, H3C 1K3, Canada; c Polytechnique Montréal, 2500 Chemin de Polytechnique Montréal (Québec), H3T 1J4, Canada; d Applied Medical Science Department, Community College, King Saud University, P.O. Box 10219, Riyadh, 11433, Saudi Arabia; e Chemical and Petroleum Engineering Department, College of Engineering, United Arab Emirates University (UAEU), P.O. Box. 15551, Al Ain, United Arab Emirates

**Keywords:** Composite drilling, Hybrid palm/jute polyester composites, Drill materials, Hole quality, Uncertainty analysis

## Abstract

This study presents a method for modelling, predicting, and evaluating the impact of drill materials on the drilling process of hybrid palm/jute polyester composites, with the aim of enhancing hole quality regarding delamination, circularity, and cylindricity. Three drill materials, including High-Speed Steel (HSS), 5 % Cobalt-coated High-Speed Steel (HSS-Co5), and Solid Carbide drills were tested, and their impacts on drilling performance were assessed. Through thorough experimentation and statistical analysis, significant differences in results were observed between HSS drills and both HSS-Co5 and Solid Carbide drills. However, the variation in results between HSS-Co5 and Solid Carbide drill results was minimal. Additionally, the findings highlight notable disparities among drill types concerning uncertainty. The results also indicate that feed rate, drill material, and their interaction play crucial roles in determining drilling efficiency. Specifically, HSS drills consistently outperformed HSS-Co5 and Solid carbide drills, demonstrating superior performance in minimizing delamination, improving circularity, and enhancing cylindricity along with lower uncertainty.

## Highlights

• Introduces predictive model for drill material impact.
• HSS drills consistently outperform alternatives.
• Drill material crucial for minimizing defects.
• Statistical analyses highlight drill material significance.

## 1 Introduction

Composite materials, owing to their exceptional mechanical properties and lightweight nature, have gained widespread attention across various industrial sectors, including aerospace, automotive, marine, and construction [[1], [2], [3]]. Among these composites, hybrid fiber-reinforced polymers (HFRPs) have emerged as promising candidates due to their enhanced mechanical performance, cost-effectiveness, and sustainability [[4], [5], [6]]. Specifically, convincing way to achieve desired material properties with least amount of environmental impact is to combine natural fibers with synthetic polymers [[7], [8], [9]]. Particular interest in using these hybrid composites for structural component fabrication has come from aerospace industry, where high strength-to-weight ratio and weight reduction are crucial factors [[10], [11], [12], [13]].

However, complex qualities of natural fibres and their varied composition provide numerous obstacles when compared to machining of composites, these characteristics vary significantly in terms of composition, alignment, and mechanical behavior [14,15]. These natural fibers, such as jute, palm, Alfa, flax or kenaf, possess inherent anisotropic characteristics, meaning their mechanical properties vary depending on direction of force application, making them particularly challenging to machine accurately and consistently [16]. This anisotropic behavior arises from hierarchical structure of natural fibers, where their properties are influenced by factors such as fiber orientation, arrangement, and intermolecular bonding [17,18]. As a result, machining these materials precisely and consistently becomes challenging due to fiber behavior. To avoid uneven cutting reactions, delamination, and other machining flaws, careful consideration is required.

This variability impacts surface roughness, where fiber orientation can cause uneven cutting responses, and contributes to delamination during machining processes. Consequently, it complicates fabrication of composite components with desired dimensional accuracy and structural integrity [[19], [20], [21], [22], [23]]. Furthermore, when dealing with hybrid composites, where multiple types of fibers or reinforcements are combined with matrix materials, complexity of machining increases exponentially [24]. This complexity arises from properties of constituent materials, resulting in non-uniform cutting responses and unpredictable machining behaviors. As a result, machining processes must be meticulously planned and executed to account for these variations, ensuring integrity and quality of final composite components [[25], [26], [27], [28]].

Among various machining processes, drilling is one of the most common operations employed in fabrication of composite structures, particularly in aerospace sector [29,30], where it is frequently needed for component assembly and fastener installation [31,32]. Achieving high-quality drilled holes in HFRPs is paramount to ensure structural integrity and long-term performance of final products [[33], [34], [35]].

The quality of drilled holes in composite materials is influenced by several factors, including properties of fibers, matrix material, cutting parameters, and type of drill used [[36], [37], [38], [39]]. In recent years, substantial study has been conducted to better understand the impact of drilling parameters on hole quality in composite materials [37,[40], [41], [42]]. Pérez-Salinas et al. [43] conducted experiments to assess impact of drilling parameters on roughness and delamination factors in composite materials, concluding that specfic carbide drill bit for Kevlar outperformed diamond inlay drill bit, supported by SEM. By using Taguchi-designed experiments to investigate the delamination behavior of glass/carbon/aramid fiber-reinforced hybrid composites, Ozsoy et al. [44] addressed delamination, critical issue in composite material drilling. They found that, in dry conditions, using low cutting velocity and feed rate can minimize delamination.

Pankaj et al. [45] investigated fabrication of partially biodegradable composites with nettle and grewia optiva fibers in epoxy, evaluating their mechanical properties and drilling performance under various conditions, revealing feed rate, spindle speed, and drill diameter significantly influence delamination in hybrid composites. Barik and Pal [46] investigated quality of drilled holes in bidirectional woven carbon fiber reinforced plastic using wavelet packets of force-torque signals. They found coated drills significantly improved surface integrity and reduced delamination and circularity errors. Xu et al. [47] analyzed effects of different cutting sequence strategies on drilling CFRP/Ti6Al4V stacks. Their study highlighted advantages of diamond-coated drills over uncoated ones in terms of lower drilling temperatures, reduced forces, and minimal tool wear. Additionally, Barik et al. [48] employed Grey Relational Analysis (GRA) to optimize drilling parameters for CFRP composites, emphasizing the significant impact of axial thrust force on delamination factor. David et al. [49] examined the effects of traditional drilling parameters on hole surface roughness in hybrid fiber composites. They found that drill diameter had greatest influence, followed by feed rate and spindle speed. Regression modeling successfully captured the interactions they observed and suggested wider range of applications for machining processes and materials. Masannan et al. [50] investigated drilling process of weaved kenaf fiber-reinforced polymeric composites, revealing significant impacts of drill bit selection and cutting parameters on delamination size and thrust force, with feed rate emerging as most influential factor across all drill bit types studied.

Lastly, Benyettou et al. [51] investigated drilling performance of bio composites reinforced with date palm fibers (CDPF) using three drill types under diverse cutting conditions. Their study, employing RSM and ANOVA, reveals that uncoated super high-speed steel drill (HSS-SUPER) induces less delamination compared to TITAN and CARBIDE coated HSS drills. Additionally, Elhadi et al. [41] examine drilling-induced damage in jute/palm date fiber-reinforced polyester hybrid composite, highlighting influence of cutting parameters on hole delamination and emphasizing superior performance of high-speed steel (HSS) drills, particularly at lower feed and rotational speeds, offering crucial insights for engineering applications and materials science.

Apart from research conducted by Elhadi et al. [41,52], few studies specifically focusing on impact of cutting conditions on drilling performance and hole quality in jute/palm fiber-reinforced hybrid composites. This study aims to fill this gap by comprehensively investigating effect of drill materials on hole quality during drilling of jute/palm fiber-reinforced hybrid composites. Three characteristics named delamination, circularity and cylindricity, which are crucial indicators of hole quality in composite materials were considered. The proposed systematic experimentation and statistical analysis provide valuable insights into machining behavior of hybrid composites and offer practical guidelines for optimizing drilling processes in automotive, aerospace and other engineering applications.

The paper is structured as follows: Section 2 outlines experimental methodology, encompassing material preparation, drilling setup, Design of Experiments (DOE), and testing procedures. In Section 3, results and findings of the study are discussed, with focus on the impact of cutting conditions and drill material on hole quality. Finally, Section 4 presents conclusions drawn from the study's findings.

## 2 Material and methods

### 2.1 Material

**Fig. 1:**
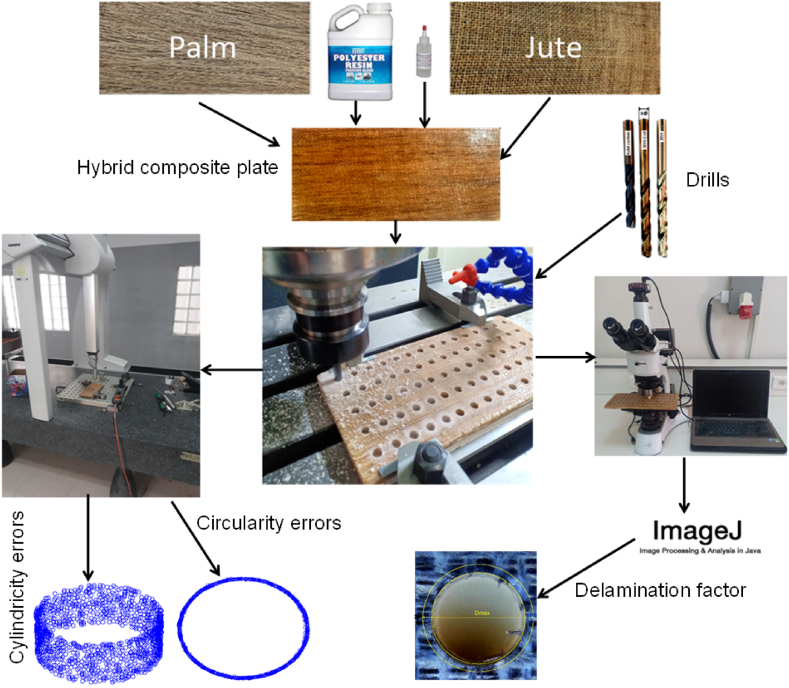
Schematic representation of the present study.

The preparation of hybrid palm/jute polyester composites commenced with methodical arrangement of dried fibers meticulously stacked in an alternating sequence to establish foundational framework. Then, precisely mixed combination of resin and hardener was meticulously spread over releasing agent layer. Employing precise hand lay-up technique, the composite was meticulously assembled, integrating 15 % jute fibers, 15 % palm fibers, and remaining constituents of polyester resin and hardener. Throughout this process, layers of unidirectional palm mat fibers underwent meticulous resin application and rolling to expel any entrapped air bubbles. Sequentially, bidirectional jute fiber layers were incorporated, and process of resin application and rolling was reiterated to ensure comprehensive removal of air bubbles. This alternating arrangement of bidirectional palm and jute fiber layers is repeated until composite attained desired thickness (Fig. 1).

Following assembly process, entire composite underwent controlled curing process at 60 °C for 8 h, ensuring proper setting. Subsequently, composite was allowed to cool under standard room temperature and pressure conditions. Once cooled, crafted composite laminates were delicately demolded, yielding samples with uniform thickness of 9 mm and boasting dimensions of $250\times 120\;{mm}^{2}$ (Fig. 1). It is noteworthy that extra precautions were taken to ensure that procedure remained consistent by carrying out each step at room temperature.

**Fig. 2:**
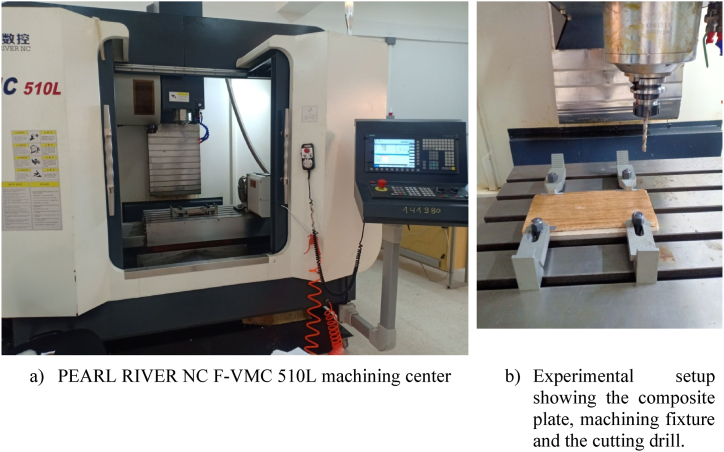
Experimental drilling setup on the machining center.

Drilling tests were conducted using state-of-the-art PEARL RIVER NC F-VMC 510L machining center (Fig. 2a). This advanced machine features latest SIEMENS 840D controllers and high-speed spindle capable of rotating at speeds up to 8000 rpm, ensuring precision and efficiency in drilling process. The complete experimental setup, which includes cutting drill, machining fixture, and composite plate, is illustrated in Fig. 2b. This setup was meticulously arranged to facilitate accurate and repeatable drilling operations.

The study employed a meticulously planned Design of Experiments (DOE) to explore effects of various drilling parameters on hole quality in composite materials. To enhance depth of investigation and facilitates comprehensive analysis, three distinct types of drills were utilized: a High-Speed Steel (HSS) twist drill (drill 1), a 5 % Cobalt-coated High-Speed Steel (HSS-Co5) twist drill (drill 2), and solid carbide (SC) twist drill (drill 3). Each drill had diameter of Ø8 mm and point angle of 135°.

The DOE focused on two main parameters: spindle speed and feed rate, with third categorical variable being drill material. The spindle speeds tested ranged from 1194 to 2786 rpm, covering broad spectrum of operational conditions to assess their impact on drilling performance. Five specific levels were selected: 1194 rpm, 1592 rpm, 1990 rpm, 2388 rpm, and 2786 rpm. Feed rates varied from 0.04 to 0.2 mm/rev, with five distinct levels: 0.04 mm/rev, 0.08 mm/rev, 0.12 mm/rev, 0.16 mm/rev, and 0.2 mm/rev, as depicted in Table 1. These feed rates were chosen to understand their influence on quality of drilled holes.

**Table 1:** Variables and respective levels used in the present study.

**Variable number**	**Variable**	**Unit**	**Variable Type**	**Level**				
				(1)	(2)	(3)	(4)	(5)
1	Speed	Rpm	Continuous	1194	1592	1990	2388	2786
2	Feed	mm/rev	Continuous	0.04	0.08	0.12	0.16	0.2
3	Drill material	–	Categorical	HSS	HSS-Co5	Solid carbide	–	–

The study examined how these factors affected errors associated to delamination, circularity, and cylindricity. Each combination of spindle speed and feed rate was tested multiple times for each type of drill material, leading to a total of 75 tests. This comprehensive approach enabled a thorough analysis of the effects on the drilled holes. Delamination measurements were taken at the entrance of the drilled holes, while circularity and cylindricity were assessed at various depths to evaluate geometric accuracy.

Owing to the sizeable dataset derived from these 75 experiments, comprehensive details about the characteristics of the drilled holes and the experimental conditions will be provided upon request.

For further access to this data, please refer to Data Availability section at the end of the manuscript. This thorough DOE approach ensures robust evaluation of drilling parameters and their impact on hole quality, providing valuable insights for optimizing drilling processes in composite materials.

The evaluation of hole quality involved precise measurements of delamination, circularity, and cylindricity using advanced instruments, including microscope and Hexagon coordinate measuring machine (CMM), as depicted in Fig. 1. The microscope was essential for detecting and quantifying delamination, while Hexagon CMM played crucial role in assessing the roundness and straightness of the hole axis. Together, these sophisticated tools ensured thorough analysis of all relevant error types, thereby upholding integrity and reliability of the components.

**Fig. 3:**
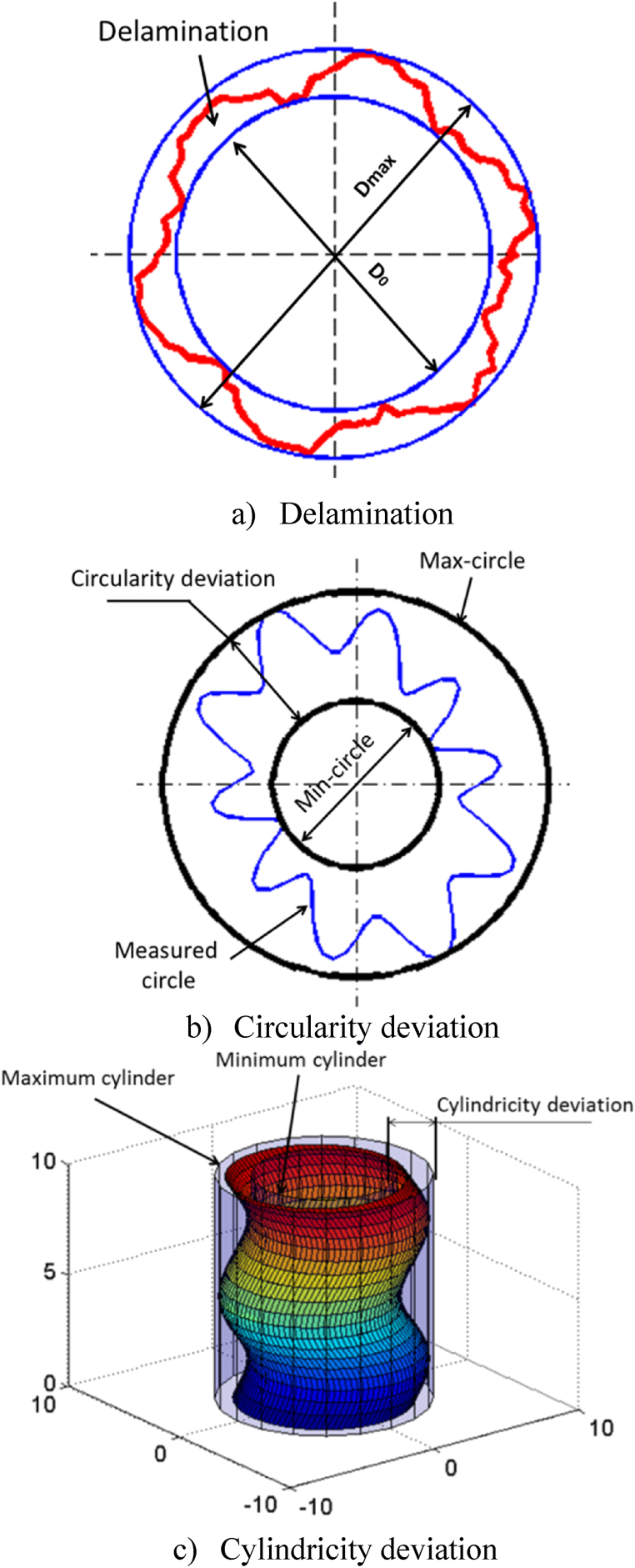
Visualization of circularity, cylindricity errors and delamination.

Delamination refers to separation of layers in composite materials around a drilled hole, typically resulting from the drilling process (Fig. 3a). This separation can significantly impact mechanical properties and structural integrity of composite. In contrast, circularity measures how closely shape of drilled hole approximates perfect circle, which is crucial for ensuring precision and quality of drilled holes, especially in composite materials, maintaining dimensional accuracy is challenging (Fig. 3b). Circularity measurements focus on the roundness of the hole, essential for components that require tight tolerances and optimal performance.

Regarding their interdependency or independency, circularity and delamination are generally considered independent characteristics. Circularity pertains to geometric accuracy of the hole, whereas, delamination concerns structural integrity of the material around the hole. However, it is worth noting that severe delamination can indirectly affect circularity if structural damage is extensive enough to alter hole's shape.

Additionally, cylindricity evaluations assess straightness of the hole's axis, ensuring precise alignment and structural integrity. Both circularity and cylindricity are important indicators of drilled hole quality, influencing overall functionality and performance of engineering structures. Consequently, thorough analysis of these metrics provides valuable insights into integrity and reliability of manufactured components, significantly contributing advancement of aerospace engineering standards and practices.

Cylindricity deviation, depicted in Fig. 3c, refers to radial differential between two coaxial cylinders within which the true surface of the component should reside. This variance must meet or fall below specified tolerance for cylindricity. Its noteworthy that cylindricity deviation represents combined form of deviation, serving to regulate both circularity and straightness of the generatrix. On the other hand, deviation in circularity can be visualized as radial gap between two concentric circles that encapsulates actual profile of the component. In Fig. 3b, illustrates by considering largest circle that fits within cross-section of the part and smallest circle that encompasses it.

**Fig. 4:**
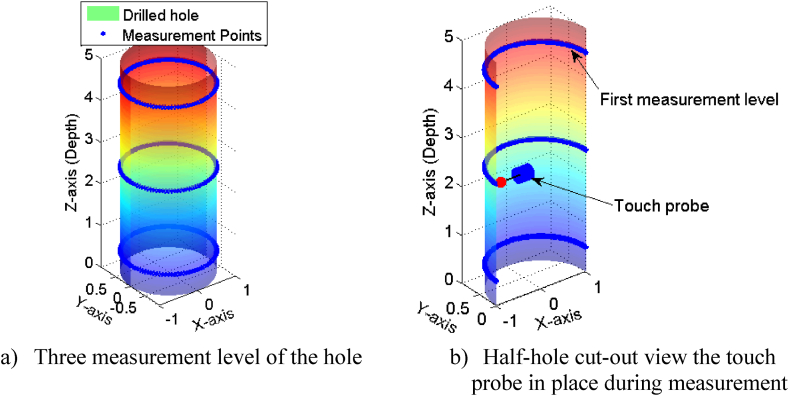
Cylindricity measurement on CMM.

As previously noted, coordinate measuring machine (CMM) was employed to acquire precise cylindricity measurements of the hole at three distinct heights (Fig. 4a). This was achieved using a high-precision probe (Fig. 4b), which enabled accurate determination of cylindricity error along the entire length of the hole.

**Fig. 5:**
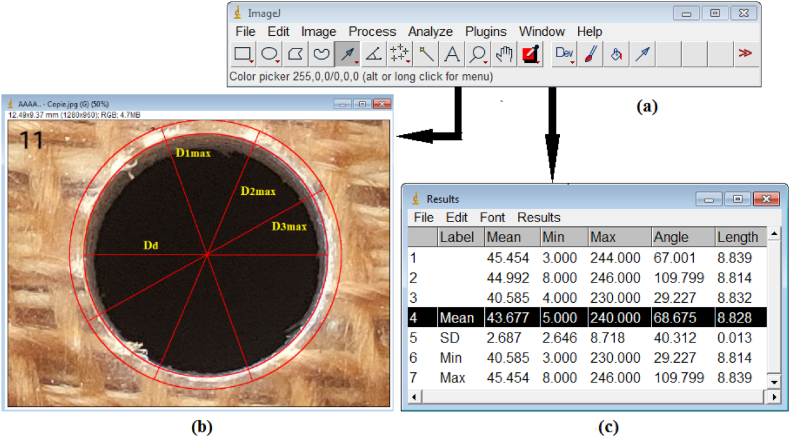
Measurement of delamination factor: a) Image J; b) Representation of the delamination zone and conjugate diameter; c) Measured values of the maximum diameter.

After drilling, delamination was clearly visible on the hole surfaces. To assess this delamination, images of the drilled holes were taken using an optical microscope and analyzed with ImageJ software. These images were imported into ImageJ (Fig. 5a), where the delaminated area was identified using the circular selection tool. This area is defined between the small circle of the drill bit and the larger circle that marks extent of the delamination (Fig. 5b). The maximum diameter of the delaminated area was measured using ImageJ. According to Chen's model [53], the delamination factor (Fd) is calculated as the ratio of the maximum diameter (Dmax) of the delaminated zone, averaged from three measurements, to the drill bit's diameter (D_0_) as shown in Fig. 3a. This model is well-regarded for quantifying delamination intensity, offering dimensionless measure of the damage. Equation (1) illustrates Chen's model:

(1) $$F_{d}=D_{\max}\backslash D_{0}$$

### 2.2 Methods

The modeling approach employed in this study involves multiple linear regression models, specifically first-order multiple linear regression model, including cross-product terms to account for interaction effects between two variables. The model is represented by Equation (2), where $Y_{i}$ is the response variable, $x_{i,1}$ and $x_{i,2}$ are predictor variables, and $\varepsilon_{i}$ represents the random error term.

(2) $${Y_{i}=\beta }_{0}+\beta_{1}x_{i,1}+\beta_{2}x_{i,2}+\beta_{3}x_{i,1}x_{i,2}+\varepsilon_{i}$$

The model is linear in its parameters $\beta_{0}$, $\beta_{1},{\;\beta }_{2}$ and $\beta_{3}$ describing a plane in the three-dimensional space of $Y_{i}$, $x_{i,1}$ and $x_{i,2}$. The parameter $\beta_{0}$ is the intercept of this plane. Parameters $\beta_{1}$, $\beta_{2}\;\text{and}\;\beta_{3}$ are referred to as partial regression coefficients.

The system of *n* equations can be expressed in matrix notation as:

(3) Y=Xβ+ε

where,

$$Y=\left[\begin{array}{c} Y_{1}\\ \vdots \\ Y_{n} \end{array}\right];\;X=\left[\begin{array}{ccc} 1 & x_{1,1} & x_{1,2}\\ \vdots & \vdots & \vdots \\ 1 & x_{n,1} & x_{n,2} \end{array}\;\begin{array}{c} x_{1,1}x_{1,2}\\ \vdots \\ x_{n,1}x_{n,2} \end{array}\right];\;\beta =\left[\begin{array}{c} \beta_{0}\\ \beta_{1}\\ \vdots \\ \beta_{k} \end{array}\right]\;\text{and}\;\varepsilon =\left[\begin{array}{c} \varepsilon_{1}\\ \vdots \\ \varepsilon_{n} \end{array}\right]$$

The matrix X is referred to as the design matrix. It contains information about the levels of the predictor variables at which the observations are obtained. The vector **β** contains all the regression coefficients.

The least square estimates are used to obtain the regression model parameters:

(4) $$\hat{\beta }={\left(X^{\prime }X\right)}^{-1}X^{\prime }Y=X^{+}Y$$

The model also accommodates qualitative factors, such as drill material, using indicator variables. In this case, an indicator variable with values 0 or 1 distinguishes between HSS drill and drills 2 or 3. The model is modified accordingly (Equation (5)), and dummy variables are tested for significance using statistical tests like the T-test, F-test, and R-squared.

(5) $${Y_{i}=\beta }_{0}+\beta_{1}x_{i,1}+\beta_{2}x_{i,2}+\beta_{3}x_{i,1}x_{i,2}+\beta_{3}D+\varepsilon_{i}$$

**Fig. 6:**
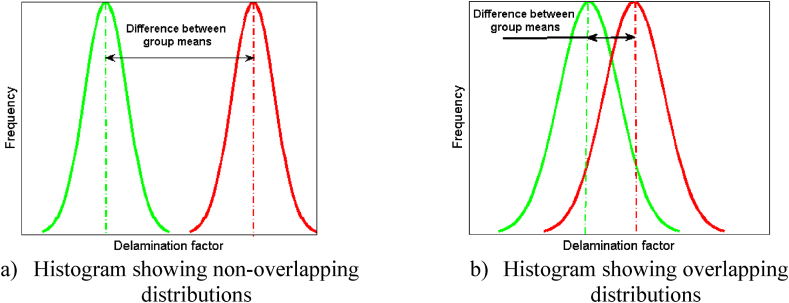
Visual representation of the *t*-test.

The Student's t-test is applied to compare mean and standard deviation, visually represented by plotting delamination factor or circularity and cylindricity errors on the X-axis and the frequency along the Y-axis of the two drills on the same graph (Fig. 6a and b). A low p-value from the T-test indicates significant effects, while the F-test assesses overall model fit. A high R-squared indicates the model's ability to explain variation in the outcome variable.

Additionally, overlap of the distributions between the two groups (HSS drill and HSS-Co5 drill or solid carbide drill) is considered. In Fig. 6a, notable differentiation between the groups is evident, indicating substantial separation in the distributions with no overlap. This distinction underscores significant discrepancy in means between the compared variables. Such clear demarcation between the distributions contributes to a higher "t" value when subjected to the *t*-test. Conversely, Fig. 6b shows high overlap of the distributions and a small difference in means, leading to a low "t" value.

In summary, this modeling approach incorporates both quantitative and qualitative factors, providing a comprehensive analysis of the relationships between predictor variables and the response variable, including an assessment of distribution overlap for deeper insights.

## 3 Results and discussion

### 3.1 Effect of cutting conditions on holes quality

Understanding the impact of cutting conditions on output responses is crucial for refining the drilling process in hybrid jute/palm polyester composites. This section delves into specific findings on how cutting conditions influence output parameters, offering insights into critical aspects for optimizing drilling in composite materials.

**Fig. 7:**
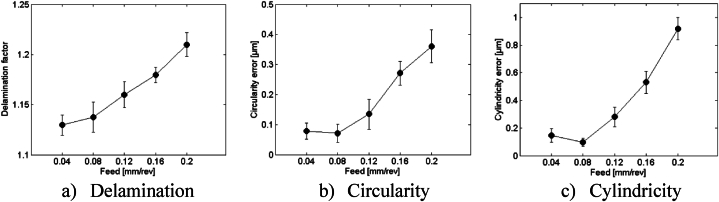
Repeatability tests at speed of 1194 rpm and different feed rates using HSS drill.

To ensure the reliability of the analysis, repeatability tests were conducted at a speed of 1194 rpm with different feed rates using HSS drill. Each test was repeated three times, measuring delamination, circularity, and cylindricity. The average values and standard deviations were calculated and are presented in Fig. 7. The mean delamination values ranged from 1.1296 to 1.21, with standard deviations ranging from 0.0075 to 0.015, indicating consistent measurements. For circularity, mean values ranged from 0.0779 to 0.36046, with standard deviations between 0.027 and 0.055. Cylindricity mean values ranged from 0.1472 to 0.9183, with standard deviations from 0.03 to 0.08. These results highlight the critical aspects of optimizing the drilling process to ensure repeatability and high-quality outputs in composite materials. The consistent measurements across repeated experiments confirm the reliability and precision of the drilling machine, ensuring confidence in the experimental findings.

Table 2, Table 3 showcase a selection of drilled holes at a speed of 1592 rpm under various cutting conditions, featuring both top and inclined views. Following this, the delamination factor, circularity, and cylindricity errors of all drilled holes were measured and analyzed.

(a) Delamination results

**Table 2:**
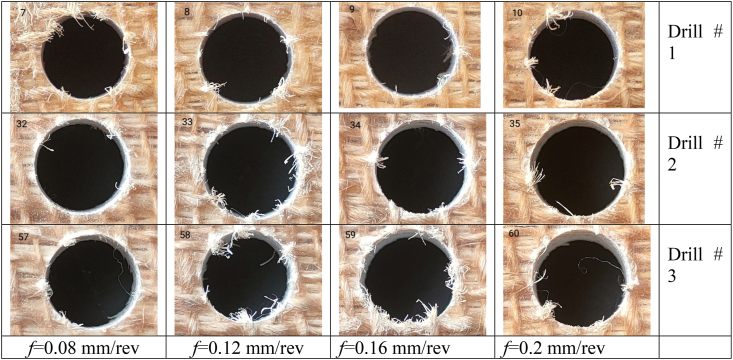
Top views of selected drilled holes at 1592 rpm with different feeds and drill Types.

**Table 3:**
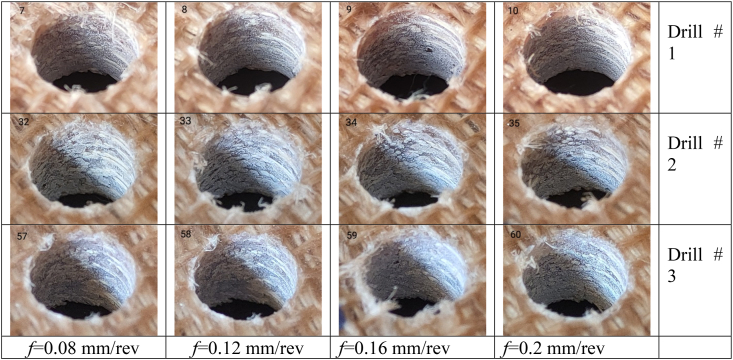
Inclined view of selected drilled holes at 1592 rpm with different feeds and drill Types.

In the analysis of delamination, regression modeling provided insights into the relationship between feed (f), speed (N), and their interaction (f×N) concerning delamination in composite material drilling. The regression model $\hat{D_{F}}$, constructed with 95 % confidence level, revealed significant contributions from both feed and speed parameters, as well as their interaction.

**Fig. 8:**
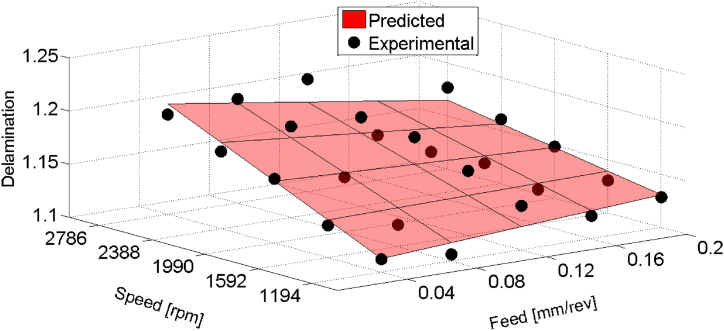
Prediction of the delamination as a function of feed and speed for HSS drill results.

The regression model for delamination factor ($\hat{D_{F}}$) was based on a first-order regression model, considering feed and speed as independent variables. Table 4 displays the statistical results for holes drilled with HSS drill, showcasing the coefficients, standard errors, F-values, and marginal contributions of each variable. The obtained regression model is shown in equation (6) and plotted in Fig. 8.

(6) $$\hat{D_{F}}=1.066+0.8303\times f+2.0025\times 10^{‐5}\times N‐0.00022\times f\times N$$

**Table 4:** Regression Model with first order interaction (HSS drill).

Significant variables	Coefficients	Standard error	F-value	Marginal Contribution	Percentage; Cont. %
Intercept	1.066	0.109			
f	0.8303	0.409	17.814	5.296	69.1
N	$2.0025\times 10^{-5}$	0.261	2.518	0.749	9.8
f×N	−0.00022	0.472	5.434	1.616	21.1

The analysis presented in Table 4 shows that Feed (f) exhibited a strong positive coefficient, indicating its substantial impact on delamination, with a contribution of 69.1 % to the total variance in delamination. This underscores the critical role of feed in minimizing delamination during drilling operations. Moreover, the interactive effect (f×N) demonstrated a notable contribution of 21.1 %, suggesting a complex interplay between feed and speed in influencing delamination. Additionally, the speed (N) contributed 9.8 % to the total variance in delamination.

ANOVA results presented in Table 5 further supported the reliability of the regression model, with an F-Fisher value of 19.83 exceeding the critical value ($F_{0.05,3,21}$ = 3.072) at a significance level of 5 %. This signifies that the regression model effectively explains the variability in delamination outcomes. The coefficient of determination ($R^{2}=0.74$) indicates that 74 % of the variance in delamination can be attributed to the regression, highlighting its predictive capability and significance in understanding delamination behavior during composite material drilling.

(b) Circularity errors results

**Table 5:** ANOVA table for the delamination factor (HSS drill).

Effect	Sum of squares	D.F.	Mean squares	F-level
Regression (SS R)	0.01369	3	0.00456	19.83
Residual (SSE)	0.004815	21	0.00023	
Total (TSS)	0.01851	24		

Moving to circularity errors, regression modeling was similarly employed to investigate relationship between cutting parameters and the circularity errors of drilled holes in composite materials. Utilizing a first-order regression model framework and a 95 % confidence level the regression model for circularity errors was also constructed based on feed (f), speed (*N*) and their interaction (f×N).

**Fig. 9:**
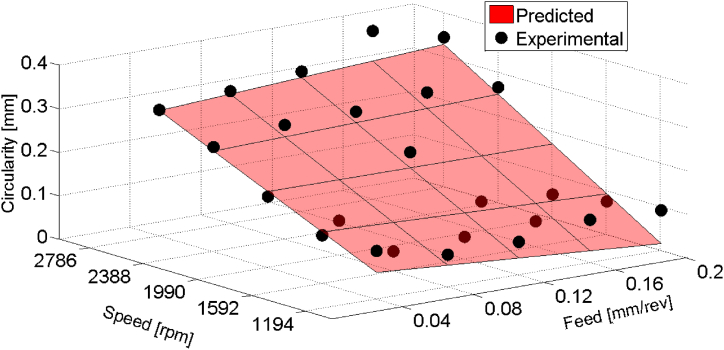
Prediction of circularity errors as a function of feed and speed for HSS drill results.

For circularity errors, the regression model $\hat{C}$ was developed (Eq. (7)). A plot of the fitted regression plane is shown in Fig. 9.

(7) $$\hat{C}=0.055+1.01\times f‐4.24\times 10^{‐5}\times N+3.35\times 10^{‐4}\times f\times N$$

Furthermore, statistical analysis for circularity errors of holes drilled with HSS drill was conducted and presented in Table 6, Table 7. Table 6 shows that feed exhibited positive coefficient with a significant contribution of 52.56 %, indicating its considerable influence on circularity errors. Additionally, speed and the interaction term contributed 22.58 % and 24.86 %, respectively, to the total variance in circularity.

**Table 6:** Regression Model for circularity errors with first order interaction (HSS drill).

Significant variables	Coefficients	Standard error	F-value	Marginal Contribution	Percentage; Cont. %
Intercept	0.055	0.102			
f	1.01	0.384	1.87	0.49	52.56
N	${-4.24\times 10}^{-5}$	0.245	0.80	0.21	22.58
f×N	${3.35\times 10}^{-4}$	0.443	0.88	0.23	24.86

**Table 7:** ANOVA table for the circularity errors (HSS drill).

Effect	Sum of squares	D.F.	Mean squares	F-level
Regression (SS R)	0.2276	3	0.0759	23.72
Residual (SSE)	0.0676	21	0.0032	
Total (TSS)	0.2952	24		

ANOVA results depicted in Table 7 confirmed the reliability of the regression model, with an F-Fisher value of 23.72 exceeding the critical value at a significance level of 5 %. The coefficient of determination ($R^{2}=0.77$) suggests that 77 % of the variance in circularity errors can be explained by the regression model.

(c) Cylindricity errors results

Regression modeling was also utilized to explore the relationship between cutting parameters and the cylindricity errors of drilled holes in composite materials. Employing the same first-order regression model framework and a 95 % confidence level, the regression model for cylindricity errors was:

(8) $$\hat{\text{Cy}}=‐0.071+3.862\times f‐6.88\times 10^{‐5}\times N+0.00045\times f\times N$$

**Fig. 10:**
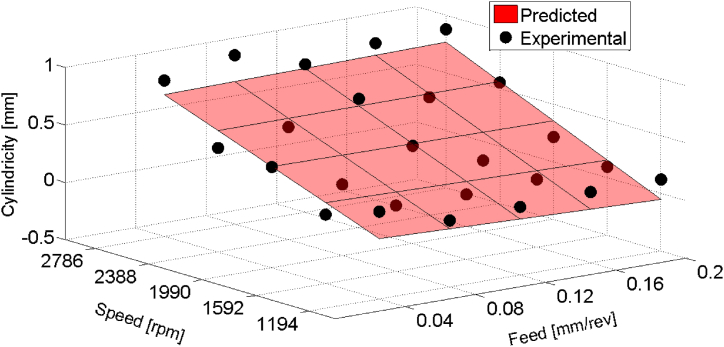
Prediction of cylindricity errors as a function of feed and speed for HSS drill results.

For cylindricity errors, the regression model ($\hat{Cy}$) was established and plotted in Fig. 10. Moreover, statistical results for holes drilled with HSS drill are shown in Table 8. Feed displayed a substantial positive coefficient, contributing 88.09 % to the total variance in cylindricity errors. Speed and the interaction term contributed 6.80 % and 5.11 %, respectively, indicating their lesser but still significant roles.

**Table 8:** Regression Model for cylindricity errors with first order interaction (HSS drill).

Significant variables	Coefficients	Standard error	F-value	Marginal Contribution	Percentage; Cont. %
Intercept	−0.071	0.094			
f	3.862	0.354	4.22	0.940	88.09
N	${-6.88\times 10}^{-5}$	0.226	0.33	0.0725	6.80
f×N	0.00045	0.408	0.24	0.0545	5.11

ANOVA results (Table 9) supported the reliability of the regression model, with an F-Fisher value of 28.98 exceeding the critical value at a significance level of 5 %. The coefficient of determination ($R^{2}=0.80$) suggests that 80 % of the variance in cylindricity errors can be attributed to the regression model.

**Table 9:** ANOVA table for the cylindricity errors (HSS drill).

Effect	Sum of squares	D.F.	Mean squares	F-level
Regression (SS R)	1.817	3	0.6058	28.98
Residual (SSE)	0.439	21	0.0209	
Total (TSS)	2.257	24		

These analyses highlight the significant impacts of feed and speed on the circularity and cylindricity errors of drilled holes in composite materials. Optimizing these parameters is crucial for achieving desired levels of circularity and cylindricity, contributing to enhanced hole quality in composite material drilling processes.

### 6.1 Effect of drill material on holes quality

(a) Comparative analysis of drill materials for enhanced performance incorporating uncertainty analysis

Uncertainty analysis is crucial in manufacturing as it quantifies variability and reliability of drilling results. In practical settings, managing this uncertainty ensures consistent and reliable drilling processes. By incorporating uncertainty analysis, variability in hole quality can be better predicted, allowing for informed adjustments that improve process stability and performance.

**Fig. 11:**
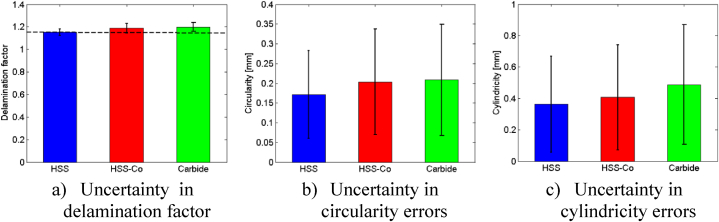
Comparison of drilling performance metrics across different drill materials.

To determine the optimal drill, an analysis of average values and standard deviations for the delamination factor, circularity, and cylindricity errors was conducted for each drill under various cutting conditions. The results indicate that HSS drill consistently outperforms the HSS-Co5 and Solid carbide drills across all three metrics, demonstrating its superiority in achieving superior drilling outcomes (Fig. 11).

Standard deviations offer insights into the variability of data points around the average, incorporating uncertainty into the analysis. A lower standard deviation suggests less variation in delamination values (Fig. 11a). The HSS drill exhibits the lowest standard deviation, indicating greater consistency in results compared to the HSS-Co5 drill and Solid carbide drill. Despite some overlap in the ranges of circularity errors between the drills, the HSS drill once again demonstrates the lowest standard deviation, signifying more consistent results (Fig. 11b).

Regarding cylindricity errors, the HSS drill showcases the lowest average (0.3628 mm), indicating superior alignment of drilled holes. Although some overlap exists in the ranges of cylindricity errors between the drills, the HSS drill generally performs better (Fig. 11c). The standard deviations underscore variability in cylindricity errors, with the HSS drill consistently exhibiting lowest deviation, signifying more consistent performance.

In general, the HSS drill consistently demonstrates superior performance in minimizing delamination, enhancing circularity, and improving cylindricity compared to the HSS-Co5 drill and Solid carbide drill. Despite some overlap in the results, particularly in circularity and cylindricity errors, the lower uncertainty associated with the HSS drill indicate more reliable and consistent outcomes. Therefore, based on this thorough analysis, the HSS drill emerges as the preferred option for achieving superior drilling outcomes. Subsequently, an advanced analysis is conducted to assess the impact of drill material on hole quality in the following section.

b) Advanced analysis

The impact of drill material on the delamination factor was examined using regression modeling, ANOVA, Student's t-test and R-squared. First-order regression models were developed, incorporating drill material as an indicator variable (D) along with feed (f), speed (N), and their interaction (f×N). The analysis focused on comparing the performance of different drills regarding delamination.

In each regression model, the coefficients of the indicator variable for drill material (D) provided information on how the delamination factor varied between different drill materials. By adding the indicator variable, equation (6) becomes:

(9) $$\hat{D_{\text{drill}2}^{\text{drill}1}}=1.1525+0.0513\times f+0.0182\times N‐0.0275\times f\times N+0.0362\times D$$

The coefficients in equation (9) are estimated using data from both drill 1 and drill 2. When D = 0, the model excludes the effect of drill 2, thereby only explaining the impact of drill 1. Conversely, when D = 1, the model incorporates the effect of drill 2. This indicates that the model represented by equation (9) can account for the effects of both, drills 1 and drill 2, providing a comprehensive understanding of how each drill material influences the delamination factor.

**Fig. 12:**
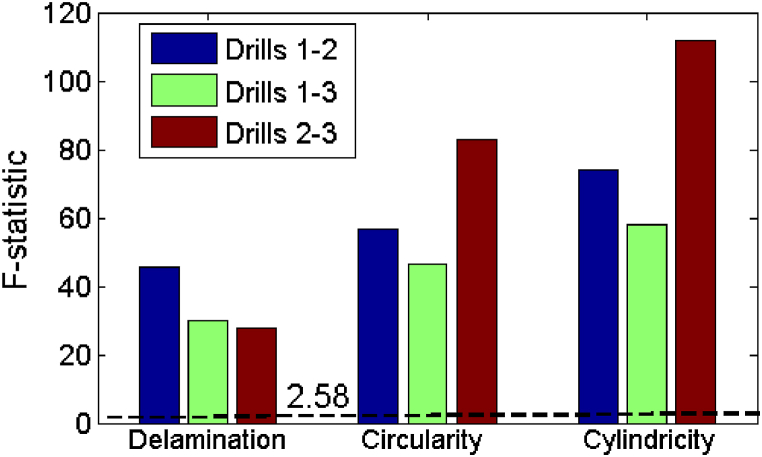
F-statistic from ANOVA results.

ANOVA test show that the model is statistically adequate since the computed F-statistic (Fig. 12) is higher than the critical F value ($F_{0.05,4,45}=$ 2.58) from the F table.

**Fig. 13:**
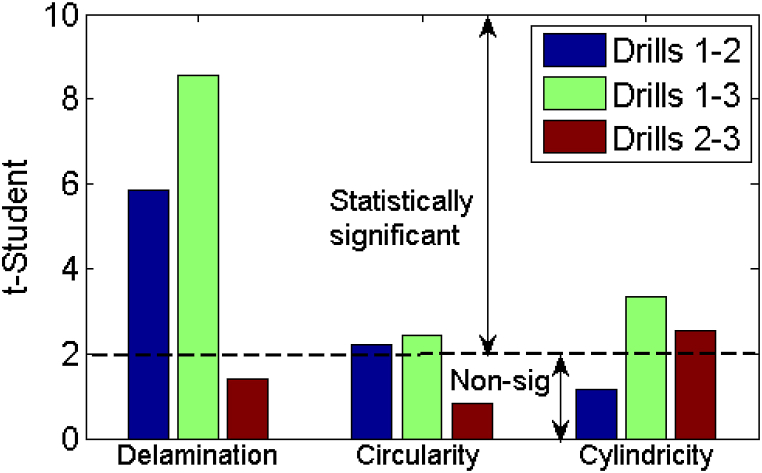
T-test results.

Furthermore, pairwise t-tests were conducted to compare the delamination factors between HSS drill and HSS-Co5 drill. These tests revealed significant effects for each drill, as indicated by the calculated t value (t = 5.85), which surpassed the critical T-value from the table ($t_{0.025;45}=2.01$). As depicted in Fig. 13, t-values (t-Student) exceeding $t_{0.025;45}=2.01$ signify the significance of the dummy variable, while t-values below this threshold indicate its non-significance.

**Fig. 14:**
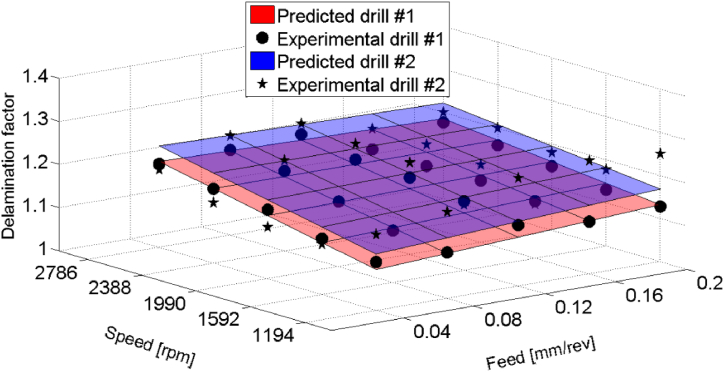
Experimental data vs. predicted linear regression planes for delamination factor related to HSS drill (D = 0) and HSS-Co5 drill (D = 1) results.

Fig. 14portrays 3D linear regression plot of equation (7), illustrating effect of the dummy variable as it transitions from D = 0 (HSS drill) to D = 1 (HSS-Co5 drill). Notably, the model demonstrates adequacy, with an R-squared value of 0.73, signifying satisfactory level of explanation for the observed variation in the delamination factor.

**Fig. 15:**
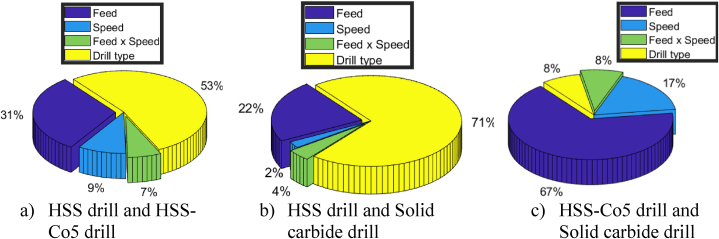
Pie chart showing percentage contributions of input parameters on delamination factor.

The analysis also assesses percentage contribution of each parameter's effect. As depicted in Fig. 15a, drill material emerges as most influential parameter affecting the delamination factor, accounting for 53 % of the variance. Following closely is the feed, which contributes 31 %, while speed and the interaction between feed and speed contribute 9 % and 7 % respectively.

Upon examining delamination factor results outlined in Table 10, it becomes apparent that regression models offer valuable insights into the correlation between drill material and delamination. In the comparison between drills 1 (HSS drill) and drill 3 (Solid carbide drill), the regression analysis revealed an R-squared value of 0.80, indicating robust relationship between the predictor variables and the delamination factor. This strong relationship is further supported by the notably high F-statistic value (F = 45.71), surpassing the critical F value ($F_{0.05,4,45}=$ 2.58). Additionally, Fig. 15 illustrates that drill material, alongside other parameters, significantly impacts delamination outcomes, contributing to 71 % of the variance, followed by feed at 22 %, the interaction between feed and speed at 4 %, and speed at 2 %. The significance of drill material is affirmed by the *t*-test results, as depicted in Fig. 13.

**Table 10:** Summary of delamination factor analysis for different drill materials.

Drills	Experimental vs. predicted values	Regression model	Model adequacy	Drill effect
1–2		$\hat{D_{drill2}^{drill1}}=1.1525+0.0513\times f+0.0182$ × N−0.0275×f×N+0.0362×	$R^{2}=0.73$	Significant
1–3		$\hat{D_{drill\;3}^{drill\;1}}=1.1525+0.0474\times f+0.0098$ × N−0.02386×f×N+0.04532×D	$R^{2}=0.80$	Significant
2–3		$\hat{D_{drill\;3}^{drill\;2}}=1.188+0.051\times f+0.016$ × N−0.021×f×N+0.009×D	$R^{2}=0.71$	Non-Significant

In contrast, when comparing drill 2 (HSS-Co5 drill) and drill 3 (Solid carbide drill), percentage contribution of drill material drops to 8 %, with the feed emerging as the dominant factor, contributing to 67 % of the variance. Speed accounts for 17 %, while the interaction between speed and feed contributes 8 % (Fig. 15c). Furthermore, regression model yielded an R-squared value of 0.71, indicating its adequacy (${F=27.69>F}_{0.05,4,45}=$ 2.58), as shown in Fig. 12. However, despite the model's adequacy, the relatively lower R-squared value suggests weaker relationship. This implies that, in this specific scenario, the influence of drill material on delamination may not be statistically significant. This conclusion is corroborated by the computed t-value (t = 1.42), which falls below the critical t-value from the table ($t_{0.025;45}=2.01$), as illustrated in Fig. 13.

In Table 11, circularity errors analysis for various drill materials is presented, focusing on the experimental versus predicted values, regression model details, model adequacy, and the impact of drill material on circularity errors.

**Table 11:** Summary of circularity errors analysis for different drill materials.

Drills	Experimental vs. predicted values	Regression model	Model adequacy	Drill effect
1–2		$\hat{C_{drill\;2}^{drill\;1}}=0.17+0.083\times f-0.0018$ × N+0.031×f×N+0.032×D	$R^{2}=0.81$	Significant
1–3		$\hat{C_{drill\;3}^{drill\;1}}=0.1714+0.093\times f-0.0103$ × N+0.027×f×N+0.037×D	$R^{2}=0.83$	Significant
2–3		$\hat{C_{drill\;3}^{drill\;2}}=0.203+0.118\times f+0.0123$ × N+0.0111×f×N+0.00531×D	$R^{2}=0.88$	Non-Significant

**Fig. 16:**
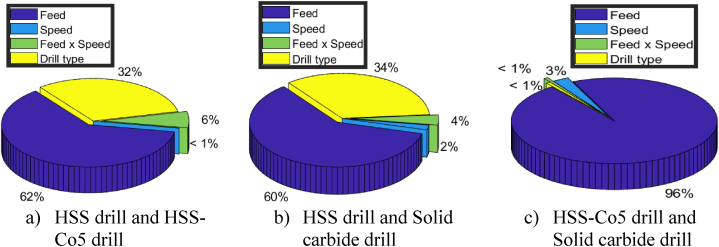
Pie chart showing percentage contributions of input parameters on circularity errors.

For the comparison between drill 1 (HSS drill) and drill 2 (HSS-Co5 drill), regression model indicates strong relationship (R-squared = 0.81), supported by significant F-statistic (F = 46.5) that exceeds the critical F-value (Fig. 12). Additionally, the *t*-test (Fig. 13) confirms significance of the drill material (t = 2.2 > $t_{0.025;45}=2.01$), indicating its substantial effect on circularity errors. On the other hand, as shown in Fig. 16a, the feed makes the most substantial contribution (62 %), followed by the drill material (32 %) and the interaction between feed and speed (6 %).

Similarly, the comparison between drill 1 (HSS drill) and drill 3 (Solid carbide drill) shows a significant relationship (R-squared = 0.83), with a high F-statistic (F = 56.65) as depicted in Fig. 12 along with a significant t-value (t = 2.43) illustrated in Fig. 13. These findings emphasize the considerable impact of drill material on circularity errors. Additionally, the analysis underscores the continued dominance of the feed factor (60 %), followed by drill material (34 %), the interaction term (4 %) and the speed (2 %) as shown in Fig. 16b.

However, in the comparison between drill 2 (HSS-Co5 drill) and drill 3 (Solid carbide drill), although the regression model displays adequacy (R-squared = 0.88) and the F-statistic is high (F = 82.92) as indicated in Fig. 12, the computed t-value (t = 0.38) shown in Fig. 13 falls below the critical t-value. This implies that in this particular scenario, the influence of drill material on circularity errors lacks statistical significance. As depicted in Fig. 16c, the feed emerges as the predominant factor (96 %), with minimal contributions from speed (3 %) and the interaction term (feed × speed) (<1 %), while the impact of drill material is negligible (<1 %).

**Fig. 17:**
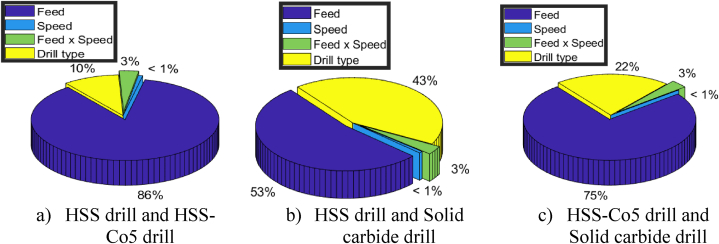
Pie chart showing percentage contributions of input parameters on cylindricity errors.

The results presented in Table 12 and Fig. 17 offer valuable insights into the relationship between different drill materials and cylindricity errors, along with their respective contributions.

**Table 12:** Summary of the statistical analysis of cylindricity errors results.

Drills	Experimental vs. predicted values	Regression model	Model adequacy	Drill effect
1–2		$\hat{{Cy}_{drill\;2}^{drill\;1}}=0.363+0.247\times f-0.015$ × N+0.056×f×N+0.044×D	$R^{2}=0.84$	Non-Significant
1–3		$\hat{{Cy}_{drill\;3}^{drill\;1}}=0.3628+0.26\times f-0.019$ × N+0.073×f×N+0.125×D	$R^{2}=0.87$	Significant
2–3		$\hat{{Cy}_{drill\;3}^{drill\;2}}=0.407+0.284\times f+0.005$ × N+0.067×f×N+0.0814×D	$R^{2}=0.91$	Significant

Table 12 illustrates the results of regression analyses comparing cylindricity errors among various drill materials. The comparison between drill 1 and drill 2 reveals substantial relationship, as indicated by an R-squared value of 0.84 and relatively high F-statistic (F = 58.05). However, the calculated t-value (t = 1.16) is below the critical threshold ($t_{0.025;45}=2.01$), implying that the impact of drill material on cylindricity errors lacks statistical significance (Fig. 13).

Conversely, when comparing drill 1 and drill 3, notable relationship emerges, supported by an R-squared value of 0.87 and bolstered by a significant F-statistic (F = 73.82). Additionally, the noteworthy t-value (t = 3.35) underscores influential role of drill material in shaping cylindricity errors (Fig. 13). Similarly, comparison between drill 2 and drill 3 also reveals significant association, characterized by an R-squared value of 0.91 and a substantial F-statistic (Fig. 12). The significant t-value (t = 2.53) further validates the impact of drill material on cylindricity errors (Fig. 13).

Moving on to Fig. 17, which provides the percentage contributions of significant variables, it is evident that drill material plays a significant role in cylindricity errors. In the comparison between drill 1 and drill 2, drill material contributes 10 % to the variance in cylindricity errors. However, the feed is the dominant factor, contributing 86 %, followed by the interaction term with 3 %. The effect speed is negligible (<1 %)

In the comparison between drill 1 and drill 3, drill material's contribution increases substantially to 43 %, indicating its significant impact on cylindricity errors. Additionally, the feed remains a significant contributor at 53 %, while the interaction term contributes 3 % and speed at (<1 %).

Similarly, in the comparison between drill 2 and drill 3, drill material contributes significantly (22 %) to the variation in cylindricity errors. The feed remains the dominant factor, contributing 75 %, followed by the interaction term with 3 % and speed at (<1 %).

Overall, research indicates that drill material indeed influences cylindricity errors significantly. However, it is noteworthy that the feed rate also plays substantial role, demonstrating higher percent contribution compared to drill material. Moreover, the interaction between feed rate and spindle speed contributes moderately to cylindricity errors. These findings emphasize the necessity of factoring in both drill material and feed rate when selecting drills and optimizing processes to reduce cylindricity errors during machining operations.

c) Enhancement of predictive accuracy through the Inclusion of quadratic terms

First-order regression models were found to be sufficient; however, their R-square values fell below 80 % in most instances. To enhance model fitness and minimize prediction error, the implementation of second-order models is recommended. The analysis indicates that incorporating quadratic terms into the regression models for delamination factors, circularity errors, and cylindricity errors significantly enhances model fitness and prediction accuracy. The revised models, which include quadratic terms, exhibit improved R-square values, ranging from 0.76 to 0.97 across different analyses (Table 13, Table 14, Table 15). This improvement is evident in the increased R-square values, which exceed 80 % in most cases, and the enhancements in prediction accuracy, ranging from 1 % to 11 %. The interaction terms, present in the original models, remain integral to capturing the complexities of the relationships between the variables. The addition of quadratic terms has further refined the models, providing more robust and accurate representation of the underlying phenomena. This approach has proven effective in capturing the intricate variations in delamination factors, circularity errors, and cylindricity errors, thereby offering more precise predictive capability for different drill materials.

**Table 13:** Summary of delamination factor analysis for different drill materials using second order model.

Drills	Experimental vs. predicted values	Regression model	Model adequacy	Improvement	Drill effect
1–2		$\hat{D_{drill\;2}^{drill\;1}}=1.1525+0.0547\times f+0.0919$ × N−0.0275×f×N− 0.0035 × $f^{2}-0.0742\times N^{2}+$ 0.0362×D	$R^{2}=0.77$	4 %	Significant
1–3		$\hat{D_{drill\;3}^{drill\;1}}=1.1525+0.0438\times f+0.0477$ × N−0.0239×f×N+ 0.0037 × $f^{2}-0.0382\times N^{2}+$ 0.0453×D	$R^{2}$ =0.81	1 %	Significant
2–3		$\hat{D_{drill\;3}^{drill\;2}}=1.188+0.0469\times f+0.0965$ × $N-0.0208\times f\times N+0.004\times f^{2}-0.0807\times V_{C}^{2}+0.009\times D$	$R^{2}=0.76$	5 %	Non-Significant

**Table 14:** Summary of circularity errors analysis for different drill materials using second order model.

Drills	Experimental vs. predicted values	Regression model	Model adequacy	Improvement	Drill effect
1–2		$\hat{C_{drill\;2}^{drill\;1}}=0.1714-0.0929\times f+0.0054$ × $N+0.0307\times f\times N++0.1798\times f^{2}-0.0072\times V_{C}^{2}+0.0319\times D$	$R^{2}=0.88$	7 %	Significant
1–3		$\hat{C_{drill\;3}^{drill\;1}}=0.1714-0.0277\times f-0.0383$ × $N+0.0267\times f\times N++0.123\times f^{2}+0.0283\times V_{C}^{2}+0.037\times D$	$R^{2}=0.87$	4 %	Significant
2–3		$\hat{C_{drill\;3}^{drill\;2}}=0.203-0.0075\times f-0.0105$ × $N+0.0111\times f\times N++0.1283\times f^{2}-0.023\times V_{C}^{2}+0.0053\times D$	$R^{2}=0.91$	3 %	Non-Significant

**Table 15:** Summary of the statistical analysis of cylindricity errors results using second order model.

Drills	Experimental vs. predicted values	Regression model	Model adequacy	Improvement	Drill effect
1–2		$\hat{{Cy}_{drill\;2}^{drill\;1}}=0.363-0.3084\times f-0.0436$ × $N+0.0562\times f\times N+0.566\times f^{2}+0.0287\times V_{C}^{2}+0.044\times D$	$R^{2}=0.95$	11 %	Non-Significant
1–3		$\hat{{Cy}_{drill\;3}^{drill\;1}}=0.3628-0.2188\times f-0.1026$ × $N+0.0732\times f\times N+0.4883\times f^{2}+0.0841\times V_{C}^{2}+0.125\times D$	$R^{2}=0.94$	7 %	Significant
2–3		$\hat{{Cy}_{drill\;3}^{drill\;2}}=0.407-0.173\times f-0.0135$ × $N+0.0673\times f\times N+0.466\times f^{2}+0.019\times V_{C}^{2}+0.0814\times D$	$R^{2}=0.97$	6 %	Significant

Based on findings presented, it is evident that both HSS-Co5 and Solid carbide drills exhibit characteristics that render them less suitable for drilling jute/palm fiber reinforced hybrid composites compared to the HSS drill. Previous research has indicated that HSS drills produce lower cutting forces on such composites [15,54]. The lower cutting force of HSS drills, coupled with their ability to efficiently cut soft materials like natural fiber composites, contributes to their superior drilling performance [15,55]. This attribute allows HSS drills to apply lower stress on the composite material, resulting in less deformation and consequently improved hole quality. In contrast, HSS-Co5 and Solid carbide drills tend to generate higher cutting forces, which can increase the stress on the material. This elevated stress may cause greater deformation, potentially exacerbating issues such as delamination and irregularities in circularity or cylindricity. However, confirming the assumption about stress and deformation differences requires measuring cutting forces and temperatures with precise instruments.

The relationship between heat generation and cutting force is crucial for understanding drilling performance of these drills. Higher cutting forces from HSS-Co5 and Solid carbide drills can result in elevated heat generation during drilling, as more energy is transferred to the workpiece. This heightened temperature can cause thermal damage to composite material, including delamination and distortion, particularly at the hole perimeter. In contrast, lower cutting force of HSS drills results in reduced heat generation, minimizing risk of thermal damage and enhancing hole quality.

Additionally, material properties of HSS-Co5 and Solid carbide drills may pose challenges in chip evacuation and cutting efficiency. HSS drills generally demonstrate superior cutting efficiency and chip evacuation capabilities compared to HSS-Co5 and Solid carbide drills. This enhanced cutting efficiency of HSS drills leads to cleaner cuts with reduced damage to the composite material, thereby improving hole quality and reducing defects like delamination and irregular circularity or cylindricity.

Finally, this study offers valuable insights into the effects of machining parameters on circularity error in composite materials; however, it does not account for the influence of tool wear on drilling performance. Tool wear is critical factor that can affect cutting forces, temperatures, and hole quality over time. In practical applications, tools experience wear, potentially impacting the accuracy and consistency of the drilling process.

Future research should aim to incorporate tool wear analysis to better understand how it effects machining parameters and create more reliable recommendations for drilling hybrid composites. Future research can improve the findings economic feasibility and practical usefulness by addressing tool wear.

## 7 Conclusion

This study provides an in-depth analysis of the drilling behavior of hybrid palm/jute polyester composites, emphasizing the ways in which drill materials and cutting parameters impact errors related to delamination, circularity, and cylindricity errors. Various statistical tools, including first and second-order regression modeling, ANOVA, Student's t-test, and R-squared analyses, were utilized to evaluate the role of drill material in drilling performance. The results indicated that both first and second-order regression models, with drill material as an indicator variable, along with feed rate, speed, and their interaction, were effective in predicting delamination, circularity, and cylindricity errors. Significantly, second-order regression model provided more nuanced understanding of the relationships between variables, capturing non-linear effects and interactions that first-order model was unable to capture. This enhanced precision underscores the critical role of detailed modeling in accurately predicting drilling outcomes.

Notably, HSS drills consistently outperformed other drill materials in terms of delamination, circularity, and cylindricity errors, exhibiting lower uncertainty and thus greater reliability and consistency in drilling outcomes. The assessments of delamination and circularity errors revealed substantial differences between HSS drills and other types, confirmed by significant percent contributions. However, when comparing HSS-Co5 and solid carbide drills, differences were minimal, indicating poorer performance of alternative drills in terms of delamination and circularity. For cylindricity errors, influence of drill material was more varied, accounting for 10 % and 43 % when comparing HSS with HSS-Co5 and solid carbide drills, respectively. The contribution of drill material (HSS-Co5 versus solid carbide) increased to 22 % for cylindricity errors, suggesting more pronounced effect in this area.

These findings emphasize importance of selecting appropriate drill material to optimize hole quality. The insights gained from this study contribute to the enhancement of drilling processes for composite materials, especially for aerospace applications where precision and reliability are paramount. The significant impact of second-order model highlights necessity of sophisticated statistical approaches in advancing our understanding and optimization of composite material drilling.

## Data availability

Data will be made available on request.

## CRediT authorship contribution statement

**Mohamed Slamani:** Writing – original draft, Investigation, Formal analysis. **Abdelmalek Elhadi:** Writing – review & editing, Investigation, Formal analysis, Data curation. **Salah Amroune:** Writing – review & editing, Supervision, Software, Resources, Project administration, Funding acquisition. **Mustapha Arslane:** Visualization, Formal analysis, Data curation. **Walid Jomaa:** Writing – review & editing, Validation. **Hassan Fouad:** Writing – review & editing, Validation, Funding acquisition. **Jean-François Chatelain:** Writing – review & editing, Visualization, Validation, Conceptualization. **Mohammad Jawaid:** Writing – review & editing, Supervision, Resources, Funding acquisition.

## Declaration of competing interest

The authors declare that they have no known competing financial interests or personal relationships that could have appeared to influence the work reported in this paper.
